# Maternal Serum Angiopoietin-Like 3 Levels in Healthy and Mild Preeclamptic Pregnant Women

**DOI:** 10.3389/fendo.2021.670357

**Published:** 2021-04-13

**Authors:** María Fernanda Garces, Haiver Antonio Rodriguez - Navarro, Julieth Daniela Buell - Acosta, Alvaro Javier Burgos - Cardenas, Roberto Franco - Vega, Luis Miguel Maldonado - Acosta, Javier Eslava - Schmalbach, Arturo José Parada - Baños, Andres Castro - Pinzon, Elizabeth Sanchez, Edith Angel - Muller, Ezequiel Lacunza, Justo P. Castaño, Carlos Dieguez, Rubén Nogueiras, Ariel Ivan Ruiz - Parra, Jorge Eduardo Caminos

**Affiliations:** ^1^ Department of Physiology, Universidad Nacional de Colombia, Bogota, Colombia; ^2^ Department of Internal Medicine Endocrine Unit, Universidad Nacional de Colombia, Bogota, Colombia; ^3^ Department of Surgery, Universidad Nacional de Colombia, Bogota, Colombia; ^4^ Department of Obstetrics and Gynecology, Universidad Nacional de Colombia, Bogota, Colombia; ^5^ Centro de Investigaciones Inmunológicas Básicas y Aplicadas (CINIBA), Facultad de Ciencias Médicas, Universidad Nacional de La Plata, La Plata, Argentina; ^6^ Maimonides Institute of Biomedical Research of Cordoba, Department of Cell Biology, University of Córdoba, Córdoba, Spain; ^7^ CIBER Physiopathology of Obesity and Nutrition (CIBERobn), Instituto de Investigaciones Sanitarias (IDIS), Department of Physiology (CIMUS), Universidad de Santiago de Compostela, Santiago de Compostela, Spain

**Keywords:** ANGPTL3, pregnancy, preeclampsia, insulin resistance, ANGPTL

## Abstract

**Objective:**

Angiopoietin-like protein 3(ANGPTL3) is an important regulator of lipoprotein metabolism in the fed state by inhibiting the enzyme lipoprotein lipase in oxidative tissues. However, the possible role of ANGPTL3 throughout gestation and its relationship with hormonal and biochemical variables are still unknown. The aim of this study was to determinate serum ANGPTL3 level in healthy non-pregnant women, during healthy and preeclamptic pregnancy and postpartum.

**Methods:**

Serum ANGPTL3 was analyzed by enzyme-linked immunosorbent assay (ELISA), in a prospective cohort of healthy pregnant women *(n = 52)* and women with mild preeclampsia *(n = 21)*, and women at three months postpartum *(n = 20)* and healthy non-pregnant women *(n = 20).* The results obtained were correlated with biochemical, hormonal and anthropometric variables and insulin resistance indices.

**Results:**

Levels of ANGPTL3 were not different between the follicular and the luteal phases of the cycle in healthy non-pregnant women. There was a significant reduction in serum ANGPTL3 levels from the first to the third trimester in healthy pregnant women compared with healthy non-pregnant and postpartum women (p <0.01). ANGPTL3 levels do not differ significantly during the three trimesters of pregnancy neither in healthy women nor in preeclamptic women. The serum levels of ANGPTL3 in women who developed preeclampsia are not statistically different from those observed in healthy pregnant women in each trimester of pregnancy. A significant lineal positive correlation was observed between serum ANGPTL3 levels and triglyceride (P =0.0186, r =0.52), very low-density lipoprotein cholesterol (P =0.0224, r =0.50), and total cholesterol levels (P =0.0220, r =0.50) in healthy non-pregnant women (P 0.05). Besides, there were no significant correlations between serum ANGPTL3 and body mass index (BMI), high-density lipoprotein cholesterol, glucose, insulin, leptin, or HOMA-IR (P >0.05)

**Conclusions:**

We describe for the first time the profile of ANGPTL3 throughout pregnancy and postpartum as well as and discussed about explore their potential contribution interactions with lipoprotein metabolism throughout pregnancy and postpartum. Thus, low levels of ANGPTL3 during pregnancy might favor lipid uptake in oxidative tissues as the main maternal energy source, while may helping to preserve glucose for use by the fetus and placenta.

## Introduction

It is well known that during pregnancy, a series of profound metabolic adaptations occur to favor and ensure fetal development and maternal survival. As a result, maternal plasma triglyceride (TG) concentrations rise significantly, 2 to 4 fold, in uncomplicated late gestation and revert to pre-pregnancy levels after delivery (1–4). These metabolic changes during pregnancy appear to be attributable to different factors such as lipoprotein lipase (LPL), estrogen, progesterone, cortisol, leptin and prolactin, among others (5). On the other hand, during the third trimester of pregnancy, high levels of human placental lactogen (hPL), decrease insulin sensitivity and increase production of catecholamines, altogether stimulating lipolysis in adipose tissue and increasing free fatty acids as substrate for endogenous synthesis of other lipoproteins in the liver (5–9).

Lipoprotein lipase (LPL) plays a fundamental role in normal lipid metabolism and energy balance, by catalyzing the hydrolysis of TG component of circulating chylomicrons and very low - density lipoprotein (VLDL-c) at the luminal surface of endothelial cells from extrahepatic tissues, to release fatty acids that can be used or stored (10–12). Additionally, it has been shown that during the first trimester of pregnancy LPL activity increases in adipose tissue whereas, in the third trimester of pregnancy this activity decreases significantly (12, 13). This situation coincides with the transformation from anabolic to catabolic condition and the increase in insulin resistance as pregnancy advances (13–16). In contrast, LPL expression and activity increase significantly in placental syncytiotrophoblast during the third trimester of pregnancy and correlate with increase of placental capacity to transport lipid for fetal growth (17, 18).

Moreover, different proteins participate in the regulation of LPL activity through posttranslational mechanisms, including the inhibitory proteins ANGPTL3, ANGPTL4 and ANGPTL8 (19, 20). ANGPTL3 is expressed mainly in the liver and its inhibitory -and irreversible- activity on LPL is developed through the ANGPTL3/ANGPTL8 complex (21–24) and is exerted mostly in the fed state in oxidative tissues, such as heart, skeletal muscle, brown adipose tissue and liver, accordingly storage of TG in the white adipose tissue (WAT) is favored (20). It is now generally accepted that the relative activity of LPL in WAT and oxidative tissues reflects a balance between the systemic effects of circulating, liver-derived ANGPTL3 and ANGPTL8 and the local effects of ANGPTL4 expression in WAT (20).

Different studies have shown that women who develop preeclampsia have high TG levels throughout pregnancy (25). Data gleaned recently have exposed the important role of ANGPTL3 in lipid metabolism by inhibiting LPL activity and therefore increasing triglycerides and other lipids. Furthermore, it was recently shown that the monoclonal antibody, Evinacumab, an ANGPTL3 inhibitor, reduced triglycerides in healthy human volunteers and in homozygous familial hypercholesterolemic individuals (26, 27). Since nothing is known about ANGPTL3 role in normal pregnancy and preeclampsia, we hypothesized that the pregnancy-induced increased in TG levels could be, at least in part, mediated by ANGPTL3. Thus, in this study we determined the profile of its serum levels in a cohort of healthy pregnant women, during the three trimesters of pregnancy and three months after delivery, and in pregnant women who developed mild preeclampsia. The correlation between serum ANGPTL3 levels and biochemical, hormonal variables, anthropometric and insulin resistance indices was investigated as well.

## Materials and Methods

### Ethical Considerations

The study was approved by the Institutional Review Board at the School of Medicine of the Universidad Nacional de Colombia and all participants signed informed consent forms. Also, the study was conducted by healthcare personnel working at the Department of Obstetrics and Gynecology of the School of Medicine of the Universidad Nacional de Colombia and Hospital de Engativá in Bogotá Colombia, between May 2012 and November 2015. Additionally, all clinical care was performed following relevant institutional, national, and international guidelines.

### Study Population

This is a case-control study nested in a prospective cohort study (*n*=465) that analyzed ANGPTL3 serum levels during three periods of pregnancy in healthy pregnant women (*n*=52), women with mild preeclampsia (*n*=21), postpartum women (*n*=20), and healthy non-pregnant women at reproductive age (*n*=20). Healthy and preeclamptic women were randomly selected from the original prospective cohort study taken into account the pregnancy outcome. Additionally, normal pregnant women and those who developed mild preeclampsia belong to the full original cohort of pregnant women.

During their first visit, maternal demographics and baseline characteristics were collected and gestational ages were determined based on last menstrual period and ultrasound during the first trimester. Additionally, maternal anthropometric, biochemical and clinical parameters were determined at the time of each routine prenatal visit scheduled. Healthy women were studied at three-time points during pregnancy: 12.14 (11.3–13.6) (1^st^ trimester), 24.60 (23.4–27.3) (2^nd^ trimester) and 34.90 (33.5–38.6) (3^rd^ trimester) weeks of gestation and three months postpartum. The inclusion criteria for healthy pregnant women were: women without pathologic antecedents, with a normal pregnancy course and outcome, that is, women with full-term delivery or cesarean section, with babies with normal Apgar score and birth weight, who did not present fetal abnormalities or malformations and women who did not develop pathologies associated with pregnancy or adverse maternal-perinatal outcomes (28).

Additionally, women with mild preeclampsia were studied in the same cohort study during 12.22 (11.2–13.1) (1^st^ trimester), 24.43 (24.0–26.0) (2^nd^ trimester) and 34.95 (34.0–37.0) (3^rd^ trimester) weeks of gestation. Mild to moderate preeclampsia may be asymptomatic and was diagnosed as preeclampsia with systolic blood pressure < 160 mmHg, or diastolic blood pressure < 110 mmHg, normal platelet count, on-elevated liver enzymes, absence of renal insufficiency, pulmonary edema, cyanosis, new-onset headaches or visual disturbances and/or right upper quadrant or epigastric pain (29). Age matched healthy non–pregnant women (BMI 18.0 – 25.0 kg/m^2^) with regular menstrual cycles, normotensive, euglycemic and with triglycerides and cholesterol levels within the normal range were included in the study (30).

Women who met any of the following criteria were excluded from the study: current smoking, alcoholism, mental illness, pre-existing heart disease, pre-existing chronic hypertension, pre-existing diabetes mellitus, gestational diabetes, autoimmune, thyroid dysfunction, chronic kidney disease, cardiac failure, hepatic failure, thyroid diseases, renal or hepatic disease, multiple pregnancy, preterm delivery (before the 34th week), preterm premature rupture of membranes, ongoing infection and miscarriage, use of approved weight lowering pharmacotherapy or patients with a history of gastric bypass and other bariatric surgery.

### Biochemical and Hormonal Analysis

Blood samples were collected and processed to yield serum on the same protocol. Thus, overnight fasting venous blood samples were drawn from all women after an overnight fast of 10 - 12 h (0700 – 0800 h). BD Vacutainer dry tubes (5 mL) were used to draw blood, and serum aliquots were stored at -80°C until assays. All the participants underwent a serum biochemical analysis of fasting insulin, blood glucose, total cholesterol, triglyceride, HDL-c and High-sensitivity C-reactive protein (hs-CRP) as described elsewhere (28). HOMA-IR index was calculated as described by Matthews et al. (31).

$$HOMA\;IR=\left(\frac{Fasting\;glucose\;(mg/dL)\times \;Fasting\;insulin\;(\mu UI/L)}{405}\right)$$

Serum specimen samples from each women participating in the cohort study have been stored appropriately (-80˚C) and thawed immediately before used in the recent ELISA assay reported here for the analysis of circulating levels of ANGPTL3. Additionally, human serum ANGPTL3 levels were measured using a commercially available ELISA kit (Catalog Number DANL30 - R&D Systems, Inc. USA) (32). The intra and inter-assay coefficients of variation (CV) were <4.1% and <8.5%, respectively. All samples were analyzed in duplicate and the mean value of the two measurements was reported. Serum levels of leptin were analyzed as described elsewhere (28).

### Statistical Analysis

Descriptive statistical analysis was performed using statistical software R (version 3.5.1). Data with normal distribution were reported as mean (±) and standard deviation (SD), while data with non-normal distribution were reported as median and interquartile range (IQ 25 - 75), but statistical testing was conducted after logarithmic transformation (33, 34). Variables with normal distribution were compared by unpaired Student’s t-test and one-way ANOVA and repeated-measured ANOVA. Additionally, a *post hoc* analysis was made among the groups. Mann-Whitney U test, Kruskal-Wallis one-way analysis of variance and Friedman test were performed for non-normally distributed variables.

Additionally, after log transformation, continuous variables not normally distributed were normally distributed and these log-transformed variables were used for correlations and linear regression analyses using Pearson or Spearman analysis to assess the association (33, 34). Using a single regression analysis, we evaluated the potential association of serum levels of ANGPTL3 with BMI, fasting glucose, triglycerides, VLDL-c, total cholesterol, HDL-c, fasting insulin, HOMA-IR and leptin levels, during each trimester of pregnancy in healthy pregnant women and healthy non–pregnant women. A *p-value* < 0.05 was considered statistically significant

## Results

Characteristics for healthy pregnant, preeclamptic, postpartum and healthy non-pregnant women are described in **Table 1**.

**Table 1:** Characteristics of healthy non-pregnant women, healthy pregnant women and preeclamptic pregnant women.

	Healthy non-pregnant women	Healthy pregnant (trimester)			Healthy women at delivery	Preeclamptic pregnant (trimester)		
		1^st^	2^nd^	3^rd^		1^st^	2^nd^	3^rd^
**N**	20	52	52	52	20	21	21	21
**Age (years)**	20	24	–	–	23	20.5	–	–
	(19-23.5)	(19.5-31)			(19-27)	(18.5-28)		
**Gestational age (weeks)**	–	12.14	24.60	34.90	–	12.22	24.43	34.95
		(11.3 - 13.6)	(23.4–27.3)	(33.5–38.6)		(11.2–13.1)	(24.0–26.0)	(34.0–37.0)
**BMI (Kg/m^2^)**	21.45	22.33	24.28	26.32	23.19	23.5	26.1	29.70
	(19.78-23.68)	(20.71-23.66)	(22.51-25.76)	(24.42-27.7)	(21.1-25.43)	(22.2-25.5)	(24.45-28.75)	(27.4-31.6)
**Fasting glucose (mg/dL)**	84.2	78	73.5	75	79	80.5	77.5	73
	(80-90.5)	(73-83)	(69-78)	(68.5-79.5)	(77-83)	(76.8-84)	(70-81)	(70-78)
**Insulin (µU/dL)**	1.90 ± 1.03	9.48 ± 4.17	10.69 ± 4.51	12.94 ± 5.64	6.60 ± 3.60	12.01 ± 4.35	15.58 ± 5.42	15.08 ± 6.32
**HOMA – IR index**	0.40 ± 0.22	1.84 ± 0.87	1.96 ± 0.86	2.42 ± 1.13	1.32 ± 0.74	2.32 ± 0.89	2.97 ± 1.07	2.81 ± 1.31
**Total cholesterol (mg/dL)**	157.86 ± 24.18	167.95 ± 31.47	219.43 ± 40.92	241.26 ± 54.74	159.67 ± 30.6	170.03 ± 30.56	219.76 ± 41.95	235.98 ± 45.70
**HDL cholesterol (mg/dL)**	52.13 ± 8.14	57.85 ± 11.49	67.74 ± 12.41	65.68 ± 11.36	48.00 ± 6.80	53.54 ± 11.72	64.54 ± 13.81	59.55 ± 17.45
**LDL cholesterol, mg/dL**	114.18 ± 26.38	113.27 ± 30.30	145.76 ± 465.85	157.65 ± 48.72	95.90 ± 29.35	117.27 ± 34.94	146.65 ± 55.48	150.43 ± 55.58
**VLDL cholesterol (mg/dL)**	14.11	21.66	34.09	50.65	14.08	24.04	33.3	46.86
	(12.2-18.35)	(17.89-25.64)	(28.58-44.85)	(40.77-59.59)	(10.22-23)	(18.31-33.41)	(26.57-41.72)	(35.4364.13)
**Triglycerides (mg/dL)**	10.55	108.3	170.45	253.25	70.4	120.4	166.5	243,7
	(61-91.75)	(89.45-128.2)	(143.15-224.25)	(203.85-297.95)	(51.5-115.7)	(91.5-135.9)	(132.85-208.6)	(187.25-321.15)
**hs-CRP (mg/L)**	1.65 ± 1.05	5.46 ± 3.10	4.70 ± 2.29	5.50 ± 3.20	3.62 ± 3.68	5.62 ± 3.44	7.43 ± 2.86	6.79 ± 3.38
**Leptin (ng/mL)**	Follicular: 16.32 ± 2.25	20.54 ± 5.17	26.80 ± 9.66	36.96 ± 11.09	–	35.15 ± 12.54	68.98 ± 32.47	91.64 ± 41.50
	Luteal: 23.02 ± 4.60							
**Fasting ANGPTL3 (ng/mL)**	Follicular 113.59 ± 18.68	78.54 ± 41.34	81.96 ± 38.44	94.10 ± 41.81	124.30 ± 36.11	79.71 ± 34.55	67.36 ± 31.62	85.31 ± 23.48
	Luteal 115.83 ± 22.36							

Data with normal distribution were reported as mean +/- standard deviation (SD) and data with non-normal distribution were reported as median and interquartile range (IQR).

BMI, Body mass index; HDL-C, High-Density Lipoprotein Cholesterol; VLDL, Very Low-Density Lipoprotein; LDL-C, low-density lipoprotein-cholesterol; hs-CRP, High-Sensitivity C-Reactive Protein.

**Figure 1:**
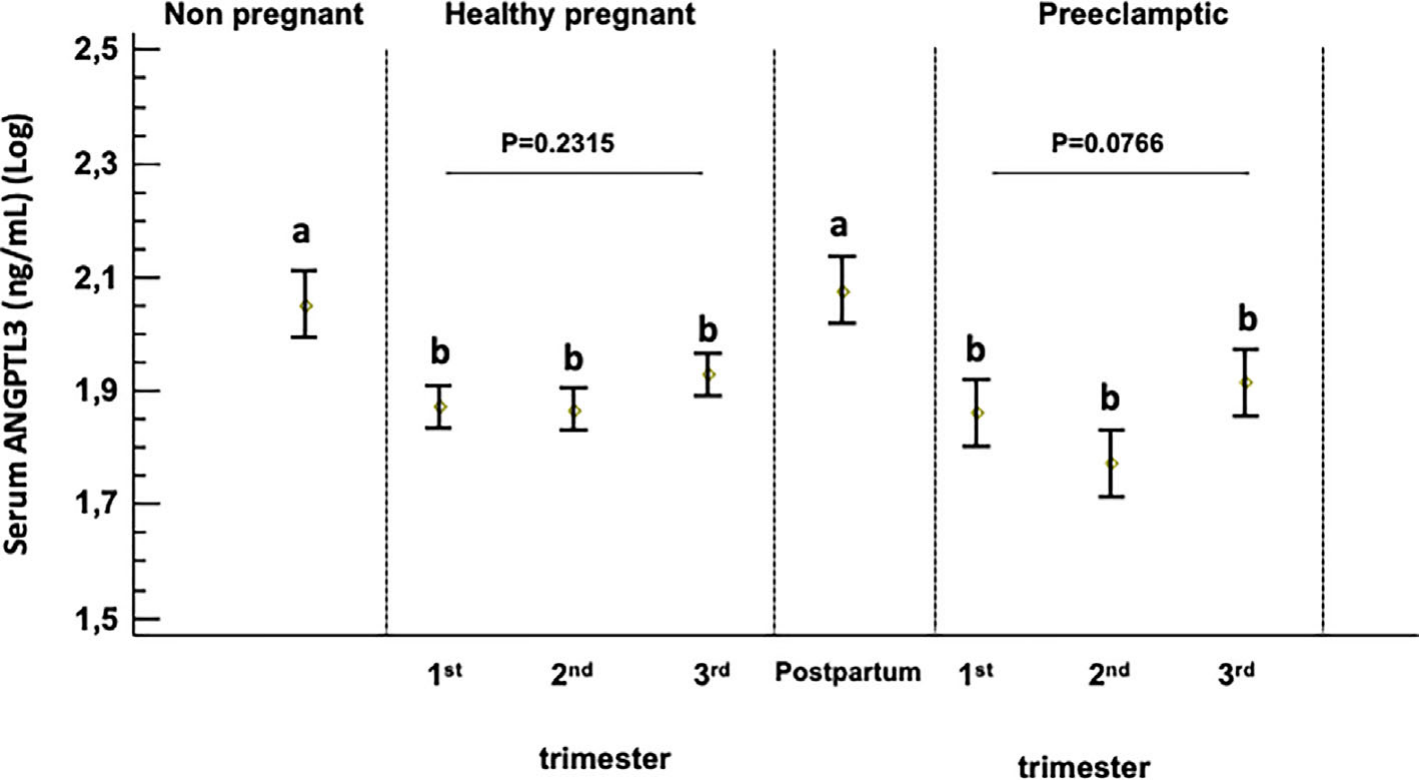
Serum ANGPTL-3 levels in healthy non-pregnant women, healthy pregnant women and women at three months postpartum and preeclamptic women. Box plot showing serum ANGPTL3 levels across pregnancy and compared with non-pregnant controls. A p-value < 0.05 was considered statistically significant. Different superscript letters (a and b) indicate which means differ significantly from which other (p<0.05) and described in statically method section. Log-transformed (log10) values were used.

Serum levels of ANGPTL3 were not different between the follicular and the luteal phases of the menstrual cycle in healthy non-pregnant women (**Table 1**). Additionally, **Figure 1** shows a significant reduction in serum ANGPTL3 levels from the first to the third trimester of pregnancy in healthy pregnant women compared with healthy non-pregnant and postpartum women (*P <*0.01).

Serum ANGPTL3 levels did not differ significantly during the three trimesters of pregnancy in healthy and preeclamptic women (**Figure 1** and **Supplementary Table 1**). In addition, serum levels of ANGPTL3 in women who developed preeclampsia are not statistically different from the levels observed in healthy pregnant women throughout the three trimesters of pregnancy studied (**Supplementary Table 2**).

**Figure 2:**
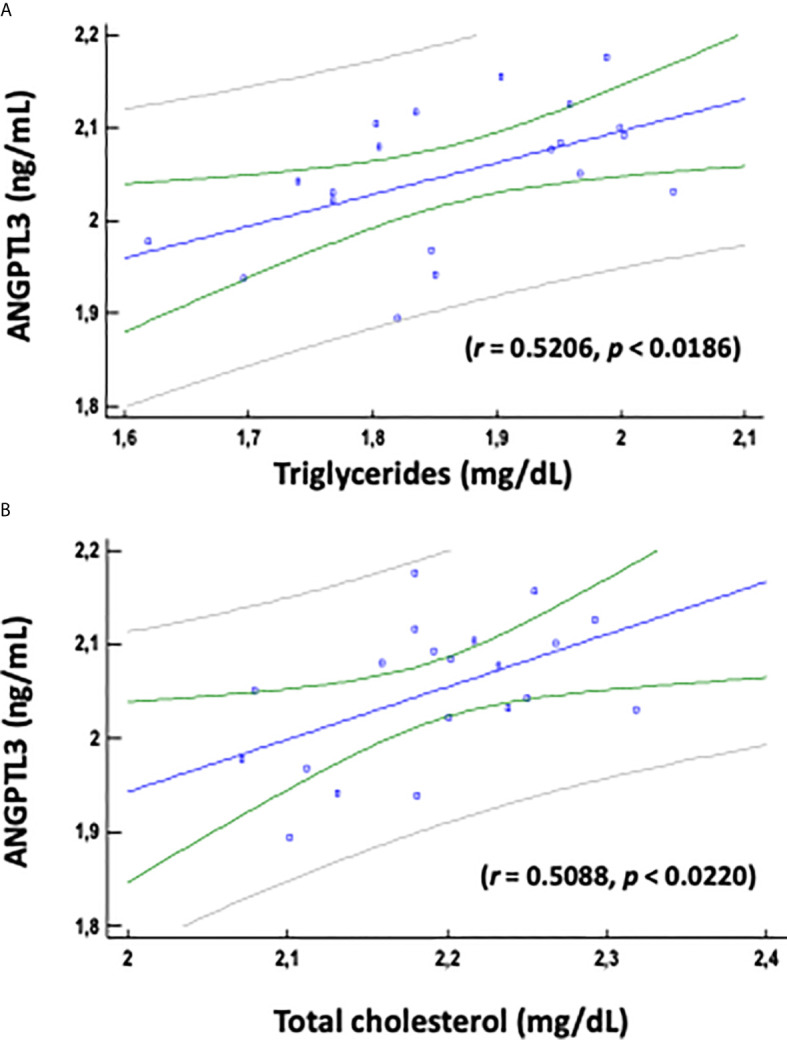
Pearson's correlation coefficient between serum ANGPTL3 levels and triglycerides **(A)** and total cholesterol **(B)** variables was determined for healthy non – pregnant women. P<0.05 (two-tailed significance). Log-transformed (log10) values were used. Linear regression presenting the associations of ANGPTL3 with triglycerides and total cholesterol.

On the other hand, correlation coefficients between ANGPTL3 serum levels and different anthropometric and metabolic parameters in healthy pregnant and healthy non-pregnant women are described in **Table 2**, **Figures 2A, B** and **Supplementary Tables 1–3**. Serum ANGPTL3 levels were positively correlated with triglycerides, VLDL-c and total cholesterol in healthy non-pregnant women (**Table 2** and **Figures 2A, B**). Conversely, serum ANGPTL3 levels were not significantly correlated with any of the biochemical variables previously described at each trimester of pregnancy in healthy pregnant women (**Supplementary Tables 1–3**).

**Table 2:** Pearson’s correlation coefficient between serum ANGPTL3 levels and study variables in healthy non – pregnant women.

Variable	Valor R	*Valor P*
BMI	-0.0825	0.7294
Triglycerides	0.5206	0.0186*
VLDL –c	0.5205	0.0186*
HDL –c	-0.0545	0.8195
Total cholesterol	0.5088	0.0220*
Glucose	-0.0874	0.7139
Insulin	0.0144	0.9518
Leptin	-0.0199	0.9336
HOMA - IR	-0.0001	0.9996

BMI, Body mass index; HDL-C, High-Density Lipoprotein Cholesterol; VLDL, Very Low-Density Lipoprotein. *P<0.05 (two-tailed significance). Log-transformed (log10) values were used.

Finally, there was not a significant relationship between serum ANGPTL3 levels and BMI, HDL-c, glucose, insulin, leptin, and HOMA-IR index in the different groups of women studied (**Table 2** and **Supplementary Tables 1-3**).

## Discussion

The present study describes for the first time, the serum profile of ANGPTL3 during the three trimesters of gestation in healthy pregnant and preeclamptic women, as well as in postpartum. Our results reveal that serum ANGPTL3 levels decreased significantly from the first to the third trimester in both healthy and preeclamptic pregnant women when compared with healthy non-pregnant and postpartum women. Additionally, there were no significant differences in serum ANGPTL3 levels between normal and preeclamptic women during each trimester of pregnancy examined in this study. Furthermore, results showed a significantly positive correlation between serum ANGPTL3 levels with triglyceride, VLDL-c and total cholesterol levels in healthy non-pregnant; however, we did not observe any significant relationship between serum ANGPTL3 and those lipids in healthy and preeclamptic pregnant women, across all trimesters of pregnancy.

Maternal accumulation of fat depots occurs in the first two trimesters of pregnancy to supply energy requirements during late pregnancy and lactation, and are attributed to different factors that can act synergistically, including increasing lipogenesis and LPL activity of adipose tissue (12–16). Furthermore, hormonal related changes during pregnancy, such as the increases of estrogen, progesterone and cortisol levels, favors endogenous *de novo* lipogenesis and fat deposition in adipose tissue (35). Previous studies have shown that adipose tissue LPL activity increases in early pregnancy or anabolic state, contributing to the accumulation of maternal fat depots during early pregnancy, and decreases as gestation progresses, favoring the adipose tissue lipolytic activity during the last third of gestation or catabolic state (35). Additionally, it has been shown that hormone-sensitive lipase (HSL) expression and activity in white adipose tissue are increased in the third trimester of pregnancy, accelerating breakdown of fat deposits and enhanced adipose tissue lipolytic activity (35). In this way, at the end of pregnancy the increased concentration of plasma non-esterified fatty acids (NEFA) and glycerol are re-esterified to triglycerides in the liver and subsequently released into the maternal circulation as native VLDL particles (12–14). As pregnancy advances, plasma triglyceride levels may raise 200 – 400% and this increase is primarily due to VLDL–TG particles (10). These VLDL-TG particles are transported to peripheral tissues, such as muscle and heart, where the triglycerides are hydrolyzed into fatty acid by action of LPL and are uptake into tissues as an important source of energy, favoring placental transfer and fetal uptake of glucose as the main energy source for the growth of the fetoplacental unit (36). In this way, it is possible that low serum ANGPTL3 concentrations profile might play a critical role during the different maternal metabolic adaptations that occur throughout pregnancy.

The results of the present study show that the serum levels of ANGPTL3 decrease significantly during the three trimesters of gestation, a condition that could contribute to the change in lipid metabolism during the anabolic and catabolic phases of pregnancy. Therefore, low levels of ANGPTL3 during the anabolic phase of pregnancy would favor the increase in *de novo* lipogenesis and the deposition of lipids due to the high activity of LPL in adipose tissue, while in oxidative tissues it would favor the activity of LPL and, therefore, the uptake of lipids as an energy source. Alternatively, during the catabolic phase of gestation, the low levels of ANGPTL3 would favor the hydrolysis of VLDL-TG and fatty acids as the main sources of energy in oxidative tissues, favoring glucose to be transported to the fetoplacental unit. In this sense, early pregnancy is an anabolic phase characterized by fat accumulation in maternal depots (2, 4, 12), while the third trimester is a catabolic condition with a breakdown of fat deposits and hyperlipidemia, and a decrease in removal the TG-rich lipoproteins from the circulation by LPL, favoring the placental lipid transport to the fetus (2, 37, 38). In this regard, previous studies have shown that ANGPTL3 is a key regulator of circulating TG levels due to its inhibitory action on the activity of LPL in the lipolysis of TG of VLDL and chylomicrons. Thus, the significant reduction in maternal circulating levels of ANGPTL3 throughout gestation could contribute that placental hormones would be the principal driving for the regulation of maternal lipids metabolism during pregnancy and these findings suggest that ANGPTL3 might be playing a reduced role in the inhibitory LPL activity during this transitory stage. Thus, the sharp decline in maternal ANGPTL3 levels throughout pregnancy might play a key role in maternal-fetal lipid metabolism adaptations.

On the other hand, serum levels of ANGPTL3 were positively and significantly associated with triglycerides, VLDL-c and total cholesterol levels in healthy non - pregnant women, which agree with previously reported results (39, 40). However, we could not find the same correlation in healthy women in any trimester of pregnancy. These data might provide potentially valuable information to further understanding of some of the features related to lipid metabolism during this critically dynamic period. In this way, these controversial relationships between ANGPTL3 levels and cholesterol and triglycerides suggest that there are other maternal factors during pregnancy that may affect the relationship between ANGPTL3 levels and other metabolic, hormonal and anthropometric factor. In addition, in controversy with previously reported results, we did not find any significant correlation between ANGPTL3 with other variables such as BMI, glucose, HDL–c, insulin, leptin and HOMA – IR index, in healthy non - pregnant and healthy pregnant women (39, 41, 42). Therefore, it is evident that assessing ANGPTL3 during pregnancy requires further investigation, in particular conducting other large prospective cohort studies associated with metabolic diseases

In conclusion, we show that in both healthy pregnant and preeclamptic women, serum ANGPTL3 levels are significantly decreased during all trimesters of pregnancy and might play a fundamental role in lipid maternal metabolism during gestation, favoring the transfer of glucose to the fetus and placenta as the main source of energy.

## Data Availability Statement

The original contributions presented in the study are included in the article/**Supplementary Material**. Further inquiries can be directed to the corresponding author.

## Ethics Statement

The study was approved by the Institutional Review Board at the School of Medicine of the Universidad Nacional de Colombia and all participants signed informed consent forms. The patients/participants provided their written informed consent to participate in this study.

## Author Contributions 

All authors contributed to the article and approved the submitted version.

## Funding

This study was supported by Government grants of The Universidad Nacional de Colombia (Dirección de investigaciones de Sede Bogotá and School of Medicine - código Hermes: 41802) and Colciencias (Departamento Administrativo de Ciencia, Tecnología e Innovación - Cod. 110154531660).

## Conflict of Interest

The authors declare that the research was conducted in the absence of any commercial or financial relationships that could be constructed as a potential conflict of interest.
