# Supplementary material for: Maternal Serum Angiopoietin-Like 3 Levels in Healthy and Mild Preeclamptic Pregnant Women
Source: Front Endocrinol (Lausanne). 2021 Apr 13;12:670357. doi: 10.3389/fendo.2021.670357 (PMC8077029; doi:10.3389/fendo.2021.670357)
Supplement: Supplementary file 1 [file DataSheet_1.pdf]

Supplementary Material

Supplementary Table 1

Variable	Valor R	Valor P
BMI	-0.1346	0.3414
Triglycerides	-0.1121	0.4289
VLDL –c	-0.1136	0.4225
HDL –c	-0.0815	0.5657
Total Cholesterol	0.0736	0.6041
Glucose	-0.1387	0.3269
Insulin	-0.0493	0.7287
Leptin	-0.1369	0.3757
HOMA - IR	-0.0689	0.6272

Supplementary Table 1. Pearson's correlation coefficient between serum ANGPTL3 levels and study variables in healthy pregnant women during the first trimester of pregnancy.

Abbreviations: BMI, Body mass index; HDL-C, High-Density Lipoprotein Cholesterol; VLDL. *P<0.05 (two-tailed significance). Log-transformed (log10) values were used.

Supplementary Table 2.

Variable	Valor R	Valor P
BMI	-0.0963	0.4969
Triglycerides	0.0506	0.7216
VLDL –c	0.0489	0.7306
HDL –c	-0.2783	0.0458
Total Cholesterol	0.1376	0.3305
Glucose	-0.2515	0.0721
Insulin	-0.0896	0.5275
Leptin	-0.1724	0.2631
HOMA - IR	-0.121103	0.3924

Supplementary Table 2. Pearson's correlation coefficient between serum ANGPTL3 levels and study variables in healthy pregnant women during the second trimester of pregnancy.

Abbreviations: BMI, Body mass index; HDL-C, High-Density Lipoprotein Cholesterol; VLDL. *P<0.05 (two-tailed significance). Log-transformed (log10) values were used.

Supplementary Table 3.

Variable	Valor R	Valor P
BMI	-0.064674	0.6487
Triglycerides	0.008855	0.9503
VLDL –c	0.006543	0.9633
HDL –c	-0.122674	0.3863
Total Cholesterol	-0.012587	0.9294
Glucose	-0.163847	0.2458
Insulin	-0.070698	0.6185
Leptin	-0.136854	0.3757
HOMA - IR	-0.092890	0.5125

Supplementary Table 3. Pearson's correlation coefficient between serum ANGPTL3 levels and study variables in healthy pregnant women during the third trimester of pregnancy.

Abbreviations: BMI, Body mass index; HDL-C, High-Density Lipoprotein Cholesterol; VLDL. *P<0.05 (two-tailed significance). Log-transformed (log10) values were used.
